# Attitude of Law and Medical Students to Oocyte Donation 

**DOI:** 10.22074/ijfs.2018.5178

**Published:** 2018-03-18

**Authors:** Samira Vesali, Elaheh Karimi, Maryam Mohammadi, Reza-Omani Samani

**Affiliations:** Department of Epidemiology and Reproductive Health, Reproductive Epidemiology Research Center, Royan Institute for Reproductive Biomedical, ACECR, Tehran, Iran

**Keywords:** Attitude, Disclosure, Infertility, Oocyte Donation

## Abstract

**Background:**

Among the young generation, medical and law students’ attitude towards third party reproduction is very important because they will be directly involved in restricting or developing the programs that will support it in the future. The aim of this survey was to investigate attitude of law and medical students to oocyte donation and key aspects of this kind of third party.

**Materials and Methods:**

In analytical cross-sectional study, 345 medical and law students were randomly selected using stratified sampling. Data was collected using attitude toward donation- oocyte (ATOD-O) questionnaire. Re- sponses were on a 5-point Likert scale. Data were analyzed according to established statistical approach by Heeren and D'Agostino.

**Results:**

The majority of the participants agreed with oocyte donation being the last choice for infertility treatment. There was a significant difference between medical students and law students regarding the acceptance of oocyte donation (3.23 vs. 3.53, P=0.025). In addition, female participants were more tolerant on receiving donated oocytes from their sisters than male participants (3.01 vs. 2.58, P=0.002) and finally, a higher number of the participants had a positive attitude towards anonymity of the donor and the recipient to one another (3.93 vs. 3.86, P=0.580). The vast majority of female students believed that the oocyte recipient naturally likes that child (P<0.0001).

**Conclusion:**

In the current study, a great majority of law and medical students support oocyte donation as an alternative way of starting a family. There is an interest among female students in donating oocytes anonymously. The majority believed that the oocyte recipient family will like the donor oocyte child naturally.

## Introduction

Since 1980s, oocyte donation (OD) has become an increasingly more accepted method of assisted reproduction, leading to a high number of OD children being born every year worldwide (1-3). Therefore, OD continues to grow in popularity and is considered as an established method to aid infertile couples in achieving their reproductive goals (4, 5). There are a few reports on OD families that have indicated no negative effects on the mother-child relationship, quality of parenting or emotional health of the children despite the absence of a genetic connection between the mother and the child (6, 7).

As the use of OD increases, so do the concerns about its psychological, social and ethical impacts on the children created in this way. Parents of OD children face important challenges during the treatment, bearing and the development of their child. These challenges include making decisions on selecting known or anonymous donors, and whether to tell others and/or the child about his or her oocyte origin (8). Not only the recipient couple, but the donor also faces emotional and social challenges regarding egg-sharing (9).

However, in recent years, particularly in developed countries, there has been a trend of couples delaying parenthood well into their fourth decade (7, 10). Subsequently, it is expected that the growing use of assisted reproductive treatments, especially third party reproduction, takes place in these countries. Among countries that are governed by Islamic law, Iran is the only country, in which third party reproduction is not illegal and any such donation as oocyte, sperm, gamete, or embryo donation, and surrogacy are currently practiced. Iran is also equipped with tourism trade (7) to meet the needs of third party treatment by couples from other countries. In addition to Iran, third party reproduction is allowed in two other countries, Azerbaijan and Turkish Republic of Northern Cyprus.

Compared to Iran, much more is known about a couple’s attitude regarding OD in the rest of the world (7, 8), but there needs to be more sufficient data on this matter in Iran. This study is aimed to investigate the attitude of law and medical students, who are directly involved in restricting or developing such reproduction programs, towards OD, and to measure the amount of their agreement on some key aspects of OD.

## Materials and Methods

A questionnaire-based study was carried out between October and December of 2014. Participants were randomly selected from medical and law students who attended in the largest university, which has two schools of law and medical together, in Tehran (capital of Iran). We used stratified sampling, in which 4^th^ year and above medical students and 3^rd^ and 4^th^ year law students were stratified by the number of years of education in each field. Then, simple random sampling was done in each stratum.

### The instrument

An analytical cross-sectional study was constructed on the basis of an earlier qualitative research on infertile couples who had referred to Royan Institute, the largest referral fertility clinic in Iran. Based on the existing literature, a pool of domains and statements was designed. 

The final version of the tool for measuring attitudes towards OD, entitled attitude toward donation-oocyte (ATOD-O), contained 52 questions in 12 domains, including the importance of having children (2 statements), decision making and acceptance of oocyte donation (7 statements), playing the role of oocyte donor (5 statements), characteristics of the donor (8 statements), characteristics of the recipient (8 statements), being an anonymous child toward the donor (4 statements), disclosure of the use of this treatment method with others (3 statements), legal issues (4 statements), tendency to the use of different methods of OD (2 statements), the parents-child relationship (4 statements), and ownership of the child (2 statements). 

For each statement, the responses were on a 5-point Likert scale, strongly disagree, disagree, somewhat, agree, and strongly agree (scoring 1 to 5, respectively). More detailed information on measuring the validity and reliability of ATOD-O questionnaire qualitatively has been published (11). To our knowledge, this tool is the first valid and comprehensive questionnaire that measures different psychological aspects of OD, with a potential for further investigations. 

### Training before the Study

It was necessary to provide information on the OD process for the participants because attitude measurement is useless without awareness about the subject. Before distribution of the questionnaires, training was conducted for about 15 to 20 minutes by a trained expert. Initially, a brief history of OD in Iran as well as in the rest of the world, was given to the students.

The process was then explained and the characteristics of potential candidate donors and recipients were described: which couples are candidates for OD (e.g., women with old age, early menopause, and who have no high quality oocytes), general characteristics of recipients and donors (donor: age from 21upto 34 years, preferably having a child/children, similar physical specifications to a recipient according to skin, eye, and hair color and the body, lack of genetic diseases even in her family history, syphilis, gonorrhea, hepatitis, and AIDS; recipient: up to 35 years, general physical examination, routine laboratory tests before pregnancy, determining the blood type and Rh, human immunodeficiency virus (HIV), hepatitis, pelvic exams, ultrasound evaluation of the uterine cavity to measure the size of the uterus, and a history of hysteroscopy, laparoscopy and spermiogram examination).

Ethical approval to conduct the study was obtained by the Ethics Committee of Royan Institute. The aims of the study were clearly explained for all participants prior to the investigation. Voluntarily filling the questionnaire was considered as consent. Participants were also made assure about their confidentiality and anonymity for attending this investigation.

### Data analysis

Statistical analyses were carried out using SPSS version 22.0 (SPSS Inc, Chicago, IL, USA). Questionnaires with missing values were not considered in the analyses. Continuous variables were expressed as mean ± SD and categorical variables as number (percentage). In this paper, responses (as 4-point Likert scale, ranging from 1 to 4) to women’s and men’s attitude and medical and law students’ attitude questions were compared using independent samples t test. Heeren and D'Agostino, in 1987, demonstrated that the t test is robust even once the outcome variable is assessed as ordinal scaled data. More details were explained elsewhere (12). P<0.05 was considered statistically significant.

## Results

### General characteristics of participants

In this study, 345 medical and law students participated. Of them, 57.7% (199 participants) were women and 42.3% (146 participants) men. About 344 participants (99.7%) were urban and 93.9% (234 participants) single. The mean age was 21.66 ± 2.07 years. Approximately 226 students (65.5%) were in medical school and 34.5% (119 participants) in law school. General characteristics of the participants are shown in Table 1.

**Table 1:** General characteristics of the participants


Demographic variables		Women n=199	Men n=146

Marital Status			
	Single	183 (92)	16 (8)
	Married	141 (96.6)	5 (3.4)
Field			
	Medicine	123 (61.8)	76 (38.2)
	Law	103 (70.5)	43 (29.5)


Values in parentheses are percentages.

### Decision making on oocyte donation

Male and female respondents were in favor of OD as the last choice of infertility treatment, while there was a significant difference between medical and law students (3.23 ± 1.18 vs. 3.53 ± 1.15, respectively, P=0.025). The difference between male and female students with regards to the recipient relationship to the donor was not significant (2.68 ± 1.17 vs. 2.67 ± 1.06, P=0.924). The difference between law and medical students with regards to supportive of relatives donating and receiving oocytes was not significant (2.58 ± 1.09 vs. 2.72 ± 1.11, P =0.275). Women were more supportive of receiving donated oocytes from their sisters compared to men. Men, on the other hand, received a question about their wife’s sister rather than their own sister (3.01 vs. 2.58, P=0.002), but in both fields there was no significant mean difference regarding this statement (2.98 ± 1.29 vs. 2.75 ± 1.30, P=0.118, Table 2).

### Anonymity and disclosure

The difference between male and female participants with regards to the statement “anonymity of the donor and the recipient to one another” was not significant (3.93 ± 1.20 vs. 3.86 ± 1.17, P=0.580). Similarly, a significant mean difference was not found between the students of the two fields. However, most of the women and the men in both fields believed that disclosure of some of the donor’s characteristics, such as age, ethnicity and religion, to the recipient couple was necessary. However, a significant difference was not observed between the groups that were compared (P=0.165 for gender groups and P=0.620 for education groups). 

Medical and law students had similar attitudes towards an OD child’s anonymity to the donor before 18 years (2.67 ± 1.24 vs. 2.55 ± 1.38, P=0.421), and there was no significant difference between men and women regarding the same statement (2.67 ± 1.30 vs. 2.57 ± 1.28, P=0.487). The mean scores obtained by the students in both genders and both fields indicated that the participants had relatively negative attitudes towards the statement “The child can meet the genetic or biologic mother after 18 years” (P=0.527 for gender groups and P=0.802 for education groups (Table 3). 

### The importance of child-parent relationship

Female participants than male respondents believed that an oocyte recipient (the mother) naturally likes that OD child (4.21 ± 0.88 vs. 3.82 ± 1.07, P<0.0001). They also believed that the husband of the oocyte recipient (the father), naturally likes the child (4.17 ± 0.91 vs. 3.79 ± 1.01, P<0.0001). Compared to the men, the women had a more positive attitude towards the two statements regarding “the child will naturally like the mother (oocyte recipient) if oocyte donation is disclosed” and “the child will naturally like the father (the husband of an oocyte recipient) if oocyte donation is disclosed” (3.95 ± 1.07 vs. 3.59 ± 1.02, P=0.002 and 4.08 ± 0.96 vs3.66 ± 1.01, P<0.0001, respectively, Table 4). 

**Table 2:** Attitudes towards decision making on oocyte donation


Statement	Women Mean ± SD	Men Mean ± SD	P value	Medicine Mean ± SD	Law Mean ± SD	P value

I am ready to use oocyte donation treatment if there are no other treatments for infertility	3.33 ± 1.20	3.36 ± 1.14	0.849	3.23 ± 1.18	3.53 ± 1.15	0.025^*^
Psychological conditions of my spouse are important for oocyte donation	4.31 ± 0.92	4.21 ± 0.99	0.364	4.23 ± 0.99	4.33 ± 0.87	0.388
I support a decision on oocyte donation by my relatives or friends	2.67 ± 1.06	2.68 ± 1.17	0.924	2.72 ± 1.11	2.58 ± 1.09	0.275
Donated oocyte from my sister is acceptable for me	3.01 ± 1.35	2.58 ± 1.20	0.002^*^	2.75 ± 1.30	2.98 ± 1.29	0.118


*; P<0.05 was considered significant statistically.

**Table 3:** Attitudes towards disclosure or secrecy of oocyte donation


Statement	Women Mean ± SD	Men Mean ± SD	P value	Medicine Mean ± SD	Law Mean ± SD	P value

The donor and the recipient should be anonymous to each other	3.93 ± 1.20	3.86 ± 1.17	0.580	3.82 ± 1.19	4.05 ± 1.16	0.097
Donor’s characteristics (such as age, ethnicity, and religion) can be given to the recipient	4.19 ± 1.02	4.04 ± 1.01	0.165	4.11 ± 1.05	4.17 ± 0.98	0.620
Recipient‘s characteristics (such as age, ethnicity, and religion) can be given to the donor	3.96 ± 1.16	3.88 ± 1.06	0.532	3.89 ± 1.11	4.00 ± 1.11	0.383
The child should be aware of his/her own genetic origin after 18 years	2.67 ± 1.30	2.57 ± 1.28	0.487	2.67 ± 1.24	2.55 ± 1.38	0.421
The child can meet the genetic or biologic mother after 18 years	2.10 ± 1.15	2.18 ± 1.51	0.527	2.15 ± 1.09	2.11 ± 1.25	0.802


**Table 4:** Attitudes towards the parent-child relationship


Statement	Women Mean ± SD	Men Mean ± SD	P value	Medicine Mean ± SD	Law Mean ± SD	P value

Oocyte recipient (the mother), naturally likes the child	4.21 ± 0.88	3.82 ± 1.07	0.000*	4.02 ± 0.94	4.08 ± 1.06	0.607
The husband of oocyte recipient(the father), naturally likes the child	4.17 ± 0.91	3.79 ± 1.01	0.000*	3.95 ± 0.95	4.12 ± 1.00	0.105
The child naturally will like the mother (oocyte recipient) if oocyte donation is disclosed	3.95 ± 1.07	3.59 ± 1.02	0.002*	3.76 ± 1.03	3.86 ± 1.12	0.408
The child naturally will like the father (the husband of oocyte recipient) if oocyte donation is disclosed	4.08 ± 0.96	3.66 ± 1.01	0.000*	3.88 ± 0.98	3.95 ± 1.04	0.498


*; P<0.05 was considered significant statistically.

## Discussion

The present findings revealed that law and medical students who participated in this study support OD as an alternative way of childbearing and starting a family. A study on attitudes of Christians and Muslims to an oocyte donation program in Iran also revealed that 74% of Christians and 59% of Muslims supported the OD for infertile couples (13).

Another survey on public opinion regarding OD in Sweden suggested that the majority of the participants believed that OD is a good way to help childless couples. However, in contrast to the findings of the present study, the Swedish women were more supportive of friends to become oocyte donors and recipients compared to the Swedish men (14). 

In the present study, the law students were significantly more in favor of this idea compared to the medical students. Interestingly, in conservative or religious societies, such as in Islamic countries, legal rights are usually consistent with the religious commands or recommendations, which are obtained from religious authorities (11). However this type of cell donation with the purpose of reproduction is not permitted to be practiced in any of the Islamic countries (15) except for in Iran-an Islamic country, which is a theocracy and is directed by the Shia laws (7). Unlike Shiites, third party reproduction is forbidden by Sunni religious scholars. However, in all Islamic countries married couples can use and benefit from assisted reproductive treatment (16).

The finding that the responders thought that the oocyte donor and the receiving couple should be anonymous (unknown) to one another was a line with earlier studies (13, 17). Although anonymity would be preferred in OD, there has been interest among donors and recipients to receive information about each other (3, 18, 19). Similar to the results of a study by Soderstrom- Anttila et al. (20), we found that some personal information, such as age, ethnicity, and religion of the oocyte donor, is an important factor in decision making by the recepient couple. 

Women also had more tendency to receiving donated oocyte from their sisters than men. Yee et al. (21), suggest that known donation will have potential challenges and problems due to an emotional relationship between the donor and the recipient. It is important to keep in mind that the feelings of both responsibility and guilt to recipient’s family appear by the donor with a family tie, especially when donor and recipient were sisters (22). The current legislation in Iran allows the donor to be anonymous for an OD procedure. 

Importantly, disclosure or non-disclosure of the genetic origin to the children is a challenging issue in OD families (23). We observed that both medical and law students had a positive attitude towards child’s anonymity to the donor before the age of 18 years. The present study confirmed the previous findings, but previous findings do not confirm a future study (6, 13, 24). At this point, a little is known about the parents’ decisions on whether to disclose their child’s origin in OD families, but there is a general agreement in favor of telling the OD children about their origins (25).

In a questionnaire-based study, Laruelle et al. (22) extracted anonymity and secrecy options of recipient couples and donors from semi-structured counseling sessions for all those who wanted to undergo oocyte donation. The participants’ motivations towards the secrecy of OD to the child were the fear of rejection by the family or their social circle and/or by the child, the fear of stigmatization by the family or their social circle, the fear of weakening the mother-child relationship, the fear of a negative psychological impact upon the child and the idea that this is intimate and does not concern the child. 

Motivations towards disclosure included matters such as to give honesty in the relationship with the child, to prevent accidental disclosure by others, to give the child potential access to his/her origins and the opportunity to meet the donor (specially the known donation group), and to avoid potential disadvantageous effects of secrecy on the parent-child relationship (23).

In a study on increasing openness in oocyte donation families regarding disclosure over 15 years, it was concluded that the professionals have more and more actively encouraged the parents to inform their presumptive children of their conceptions (3). Nonetheless, the nature of counseling provided by the medical team and psychologists is the most important factor affecting disclosure decisions (3, 26).

Surprisingly, in this study, women valued parenthood more than men and they believed that the OD child will naturally like her/his family member (the mother and the father or recipient couple), even if oocyte donation is disclosed. Since the participants of the current study were from two schools in the country, a more extensive study will on both law and medical students will help in achieving more comprehensive results.

## Conclusion

This was the first report on the attitudes of medical and law students towards OD in a Muslim country. The present findings indicated that a great majority of law and medical students support OD as an alternative way of starting a family. There is an interest amongst female students in donating oocytes anonymously. The majority of the participants believed in the importance of the relationship between parents and their child. They were concerned about the oocyte recipient family loving their OD child and vice versa naturally. We truly believe that these studies may potentially influence the law and medical students in a positive manner. 
